# Trimethoprim/Sulfamethoxazole-Induced Bradycardia, Renal Failure, AV-Node Blockers, Shock and Hyperkalemia Syndrome

**DOI:** 10.5811/cpcem.2019.5.43118

**Published:** 2019-07-22

**Authors:** Nnaemeka Diribe, Jacqueline Le

**Affiliations:** Desert Regional Medical Center, Department of Emergency Medicine, Palm Springs, California

## Abstract

BRASH (bradycardia, renal failure, atrioventricular-node blockers, shock, and hyperkalemia) syndrome is a recently coined term for a condition that describes the severe bradycardia and shock associated with hyperkalemia in patients on atrioventricular (AV)-node blocking agents. The proposed pathophysiology involves a precipitating event that exacerbates renal dysfunction with resulting AV-node blocker and potassium accumulation that act synergistically to precipitate bradycardia and hypotension. This syndrome may be refractory to the usual management of bradycardia. This case describes BRASH syndrome precipitated by trimethoprim/sulfamethoxazole.

## INTRODUCTION

Severe hyperkalemia can present as reversible bradycardia that mimics atrioventricular (AV) block.^1^ However, hyperkalemia in the setting of concomitant AV-node blocker usage can precipitate refractory bradycardia and hemodynamic instability even without a critically elevated potassium level or sizeable ingestion of AV-node blocking agents. This syndrome termed BRASH – bradycardia, renal failure, AV-node blockers, shock and hyperkalemia –is a cycle of synergy between hyperkalemia and AV-blockade that can result in cardiovascular collapse.^2^ It would generally be expected that the prevalence of such a condition be higher among the elderly who may be more likely to be on AV-node blocking medications and have a greater likelihood of events that precipitate renal failure.^3^ We report a case of BRASH syndrome occurring in a relatively young patient following a course of trimethoprim/sulfamethoxazole (TMP/SMX).

## CASE REPORT

A 51-year-old male with past medical history of pituitary carcinoma with resection, metastasis to the liver, Cushing’s syndrome, hypertension, hyperlipidemia, hypothyroidism, insulin dependent diabetes mellitus, and renal insufficiency presented to the emergency department (ED) after a syncopal episode at the office of his primary care physician (PCP). He was found to have a pulse of 20 beats per minute (bpm) and blood pressure of 60/30 millimeters of mercury (mmHg) by paramedics who immediately initiated transcutaneous pacing and transported him to the ED. On arrival, the patient was somnolent but arousable, paced at 90 bpm at 70 milliamps, and persistently hypotensive with blood pressure of 80/60 mm Hg. Pacer capture was poor. His electrocardiogram (ECG) revealed third-degree AV block and marked bradycardia with heart rate of 39 bpm, peaked T waves, and widened QRS of 173 milliseconds (Image). The patient’s bradycardia was refractory to atropine, and pacing was resumed. Initial laboratory data was significant for elevated serum potassium of 8.6 millimoles per liter (mmol/L) (reference range 3.5 – 4.9 mmol/L), blood urea nitrogen (BUN) of 51 milligrams per deciliter (mg/dL) (reference range 8 – 26 mg/dL), and creatinine of 3.3 mg/dL (reference range 0.6 – 1.3 mg/dL).

According to his PCP, his baseline creatinine level was 1.7 mg/dL. Sodium was low at 130 mmol/L (reference range 138 – 146 mmol/L), and troponin I was normal at 0.010 ng/mL (reference range 0.000 – 0.080ng/mL). Intravenous (IV) calcium chloride, insulin with dextrose, and albuterol were administered to treat his hyperkalemia. IV hydrocortisone was additionally given for potential adrenal crisis. With treatment, his heart rate and blood pressure improved to 97 bpm and 140/52 mmHg, respectively. His level of consciousness likewise was restored. Nephrology was contacted for possible dialysis, and the patient was admitted to the intensive care unit.

It was later discovered that the patient’s medications included the beta-blocker carvedilol (6.25 mg twice daily) and eplerenone (25 mg daily), a potassium-sparing diuretic. He denied any recent dosage changes or attempted overdose. Interestingly, he had been started on TMP/SMX for otitis media a week prior to presentation and had developed progressive weakness and fatigue three days after his first dose. He was being evaluated on an outpatient basis for said weakness when he had the syncopal episode. His hospital course consisted of continued therapy with additional diuresis and sodium polystyrene sulfonate, resulting in the down trending of serum potassium levels and improvement of his renal function and urine output. Within 24 hours, his potassium level corrected to 5.0 mmol/L, and therefore dialysis was not initiated. Upon discharge three days later, his potassium was 4.1 mmol/L, BUN 31 mg/dL, and creatinine 1.4 mg/dL. He was instructed to stop taking his eplerenone and was discharged in stable condition with scheduled outpatient follow-up.

**CPC-EM Capsule**

What do we already know about this clinical entity?

Atrioventricular-node blockers and hyperkalemia may act in synergy to precipitate bradycardia and shock that may be refractory to usual individual therapy.

What makes this presentation of disease reportable?

This case highlights bradycardia, renal failure, AV-node blockers, shock and hyperkalemia (BRASH) syndrome and the potentially grave risk associated with prescribing medications that can cause hyperkalemia.

What is the major learning point?

Bradycardia and shock from BRASH syndrome require intervention at multiple physiologic fronts that individually may not be grossly abnormal.

How might this improve emergency medicine practice?

Algorithmic and heuristic recognition of this syndrome could aid rapid intervention by the emergency physician and improve potential morbidity and mortality.

## DISCUSSION

Hyperkalemia is a well-known reversible cause of heart block, arrhythmia, and syncope.^3^ It can be precipitated and worsened by multiple factors including medications, renal failure, mineralocorticoid deficiency, tissue necrosis, metabolic derangement and exogenous intake.^4^ Risk factors for medication-induced hyperkalemia include diabetes mellitus, renal insufficiency, hypoaldosteronism, and age greater than 60 years.^5^ There have been multiple documented cases of hyperkalemia precipitated by TMP/SMX as sole therapy^6^,^7^ or in combination with other medications.^8^ In fact, hyperkalemia and acute renal dysfunction have been documented with onset after therapy initiation in as quickly as 24 hours, particularly in patients with baseline renal insufficiency.^9^ This is due to the direct activity of the trimethoprim component of TMP/SMX in inhibiting potassium excretion by reversibly disrupting sodium reabsorption by cells in the distal nephron.^10^

There may also be elements of renal toxicity driven by sulfamethoxazole crystal deposition.^11^ Moreover, eplerenone is an aldosterone antagonist that inhibits renal secretion of potassium and sodium reabsorption, whereas carvedilol is a beta blocker that suppresses catecholamine-driven renin release while impairing cellular potassium uptake.^4^ Our patient was therefore on several prescribed medications with the potential to drive and worsen hyperkalemia. Particularly interesting is the notion that beta blockers can act in synergy with hyperkalemia to induce bradycardia even with only mild hyperkalemia present.^3^ This is achieved by the dual role beta blockers play as direct AV-node blockers in addition to maintaining higher serum potassium levels by reducing potassium excretion and impairing cellular uptake of serum potassium.^4^ This cycle is propagated as bradycardia worsens renal perfusion leading to a spiral of worsening hyperkalemia, renal failure and bradycardic shock, which can sometimes be refractory to usual therapy.^2^

Given the pathophysiology described above, it is reasonable to expect that the population most susceptible to this syndrome would include the elderly who are often on multiple medications with potential to block the AV node or depress renal function, as well as patients with baseline renal insufficiency or heart block.^3^,^4^ It is important to note that patients presenting with BRASH syndrome may be adherent to the prescribed doses of the AV-node blocker and have not ingested additional significant quantities of the medication. There may be, however, a precipitating event such as the addition of a new medication with the potential to cause hypovolemia or exacerbate baseline renal insufficiency. In this case, the initiation of antibiotic therapy with TMP/SMX worsened our patient’s already-known renal insufficiency and on top of the eplerenone and carvedilol he was taking, contributed to his overall marked hyperkalemia and bradycardia. Furthermore, a stress corticosteroid was not used during the treatment of his ear infection.

Management of BRASH syndrome involves supportive care, treatment of shock, and expeditious correction of electrolyte abnormalities (Figure). As is standard with severe symptomatic hyperkalemia, membrane stabilization must be initiated with IV administration of calcium, insulin and dextrose, beta-agonist therapy and diuresis or dialysis as needed. Volume status must be evaluated carefully and addressed since acutely anuric patients can experience fluid overload while others may be fluid depleted. Patients may require support with vasopressors if shock remains despite other therapies, in which case epinephrine and isoproterenol could be reasonable choices given their beta-receptor activity.^2^

## CONCLUSION

BRASH – bradycardia, renal failure, AV-node blockers, shock and hyperkalemia – is a syndrome of severe bradycardic shock that is likely propagated by a synergy between AV-node blockade and hyperkalemia. The patients are generally on only prescribed doses of the AV-node blocker and present with severe bradycardia and hypotension after a precipitating event that worsens renal function. In some cases, such as the one presented here, the shock is severe enough to cause syncope. Mindfulness of the populations most likely to develop BRASH syndrome is recommended to help avoid prescribing medications that can potentially worsen renal function and predispose an already vulnerable patient to this syndrome. Immediate recognition of BRASH syndrome is likewise imperative to ensure prompt and aggressive management.

## Figures and Tables

**Image:**
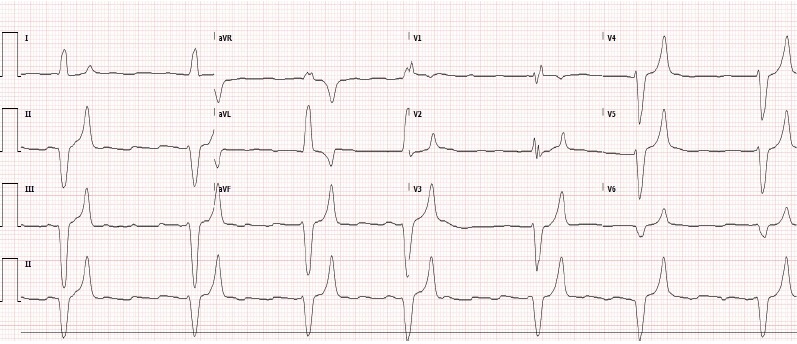
Initial electrocardiogram of 51-year-old male showing third-degree atrioventricular block with a ventricular escape rhythm. There is marked bradycardia (39 beats/minute), peaked T waves, and widened QRS (173 milliseconds).

**Figure:**
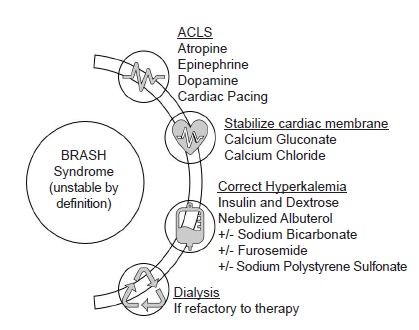
Management considerations for BRASH – bradycardia, renal failure, atrioventricular-node blockers, shock, and hyperkalemia – syndrome.
